# Effect of Day 3 and Day 5/6 Embryo Quality on the Reproductive Outcomes in the Single Vitrified Embryo Transfer Cycles

**DOI:** 10.3389/fendo.2021.641623

**Published:** 2021-04-23

**Authors:** Ningling Wang, Xinxi Zhao, Meng Ma, Qianqian Zhu, Yao Wang

**Affiliations:** Department of Assisted Reproduction, Shanghai Ninth People’s Hospital, School of Medicine, JiaoTong University, Shanghai, China

**Keywords:** birth weight, blastocyst transfer, embryo quality, live birth, vitrification

## Abstract

**Objective:**

To explore the live birth rate and neonatal outcome after single vitrified blastocyst transfer versus single vitrified cleavage-stage embryo transfer at different grades of embryo quality.

**Methods:**

A retrospective cohort study including 6077 single vitrified-thawed embryo transfer cycles was performed in the time-period from January 2013 to December 2018.

**Results:**

After controlling for potential confounding variables, there are 161% increased odds of a live birth after transfer of single good quality embryo at day 5, 152% increased odds of a live birth after transfer of single poor quality embryo at day 5, 60% increased odds of a live birth after transfer of single good quality embryo at day 6 compared with transfer of single good quality embryo at day 3. Results from the generalized estimated equation regression showed significant relationship of unadjusted birth weight with development stage of embryo and embryo quality (good quality embryo on day 5 vs. Good quality embryo on day 3:β=108.55, SE=34.89, P=0.002; good quality embryo on day 6 vs. Good quality embryo on day 3:β=68.80, SE=33.75, P=0.041). However, no significant differences were seen in birth weight between transfer single poor quality embryo on day 5, 6 and transfer single good quality embryo on day 3.

**Conclusion:**

A significant increase in live birth rate and birth weight after transfer of single good quality embryo on day 5 and day 6 compared with transfer of single good quality embryo on day 3 in the vitrified embryo transfer cycles.

## Introduction

Assisted reproductive technology (ART) brings the hope of conceiving their own child for many infertile couples, and more than seven million babies have been born through ART around the world. Many changes have been made in the clinical practice for the forty years since the first birth of *in vitro* fertilization (IVF) infant. Multiple embryo transfer was preferred in the earlier years of the clinical ART treatment. Although improving the pregnancy rate and live birth rate, the transfer of multiple embryos also result in the multiple births, which are the commonest complication associated with ART. Multiple births are significantly related with increased adverse health outcomes for both mothers and neonates, including maternal and neonatal mortality, preeclampsia, pregnancy-induced hypertension and diabetes, preterm delivery, low birth weight, intrauterine growth restriction, and prematurity (1, 2).

With the improvement of clinical and laboratory technologies, the ultimate goal of ART has changed from achieving successful pregnancy to having a healthy singleton infant born at full-term gestation. So single embryo transfer (SET) as the simplest way of curbing the multiple births are becoming increasingly common and even mandated in many countries (3).

The improvement of embryo culture technique and the widely utilization of vitrification have significantly increased the proportion of vitrified blastocyst transfer in clinical treatments. It has been reported that vitrified blastocyst transfer increased the live birth rate and significantly affected the newborn birth weight (4, 5). However, existing research did not consider the effect of embryo quality. Embryo quality, based on morphological parameters, is a major predictor for the success of implantation and live birth (6, 7). Previous studies have reported good embryo quality was strongly associated with increasing implantation rate, clinical pregnancy rate, and live birth rate (8, 9). The study performed by Ebner et al. showed a significantly higher percentage of congenital malformations among pregnancies conceived after poor quality embryo transfer (10). Whether the significant differences in pregnancy outcomes and neonatal outcomes between blastocyst transfer and cleavage-stage embryo transfer were related with embryo quality? Whether the clinical outcomes after the poor quality blastocyst transfer were superior to those after good quality cleavage-stage embryo transfer? No research has been reported in this regard.

Therefore, the aim of this study was to compare the live birth rate and neonatal outcomes after single vitrified blastocyst transfer with single vitrified cleavage-stage embryo transfer at different grades of embryo quality.

## Materials and Methods

### Study Design

A retrospective cohort study comparing clinical outcomes after vitrified blastocyst transfer with vitrified cleavage-stage embryo transfer was performed in the time-period from January 2013 to December 2018 in the Shanghai Ninth People’s Hospital affiliated to JiaoTong University School of Medicine (a large hospital-based tertiary care reproductive center in Shanghai, China). Only single vitrified blastocyst or cleavage-stage embryo transfer cycles were included. Cycles with donor oocytes or preimplantation genetic diagnosis (PGD) were not included. Mothers with pregnancy complications were excluded from this analysis.

Six study groups were compared according to the development stage of embryo and embryo quality: (1) single good quality embryo on day 3, (2) single poor quality embryo on day 3, (3) single good quality embryo on day 5, (4) single poor quality embryo on day 5, (5) single good quality embryo on day 6, (6) single poor quality embryo on day 6. This study protocol was approved by the Ethics Committee (Institutional Review Board) of the Shanghai Ninth People’s Hospital.

### Procedures

The details about ovulation induction and IVF/ICSI procedure, embryo culture and evaluation, freezing and warming of embryos, and frozen embryo transfer all have been detailed in our previous papers (11–14). IVF or ICSI was performed depending on the semen quality. Normal fertilization was assessed 16-18 hours after insemination/injection. Then the embryos were subsequently cultured until day 3 or day 5/6. The extended culture of embryos was determined according to the quantity and quality of cleavage-stage embryos, combined with the request of patients and the evaluation of clinicians.

Cleavage embryos were classified as top quality embryos if they had six to eight cells on day 3, with less than 20% anucleate fragments according to the Cummin’s criteria (15, 16). Embryos graded III or IV including those had less than six cells on day 3 and no less than 20% fragmentation were called poor quality. Blastocysts quality was graded on day 5 or 6 according to the degree of blastocoel expansion, inner cell mass (ICM), and trophectoderm (TE) cells (17). Good quality embryos were defined as those where at least: [3] the blastocele filling completely 100% of the embryo, [B] loosely grouped with several cells and [B] several cells formed in loose epithelium. Blastocysts of grade≥3BB on Day 5 or 6 were defined as good quality embryos. Embryo grading was done by two embryologists trained according to the Cummin’s criteria, and was verified by another senior embryologist with over ten years of work experience. To avoid intra-operator and inter-clinic variability in our assessment of embryos, we annually perform an internal validation. Briefly, we individually grade embryos at all stages of development and of various grades. The results are then summarized and discussed to ensure that we are homogenous in our assessment of these embryos both within the group and in relation to other clinics.

After embryos grading, all cleavage stage embryos and blastocysts were frozen using the vitrification method. In brief, the cryotop carrier system (Kitazato Biopharma Co. Ltd, Japan) was used for vitrification and 15% (v/v) ethylene glycol, 15% (v/v) dimethylsulphoxide and 0.5 mol/l sucrose was used as the cryoprotectant. For warming, 1.0 mol/l, 0.5 mol/l and 0.0 mol/l sucrose solutions were used for stepwise cryoprotectant dilution. All vitrification and warming steps were carried out at room temperature except the first warming step, which was at 37°C. The same vitrification method was employed throughout the whole study period. There were 4 vitrification operators involved in the study, all of whom have been well trained to perform vitrification technically as described in our previous study (18).

Endometrial preparation was performed as previously described (13). Natural cycle was used for patients with regular menstrual cycles, and hormone therapy cycle or stimulation cycle was used for patients with irregular menstrual cycles. Embryo transfer was conducted under ultrasound guidance, and the serum beta-HCG level was measured 14 days after embryo transfer.

### Clinical Outcomes

The primary outcome was the live birth, which was defined as an infant born alive after 24 weeks of gestation who survived more than 28 days (19). The clinical pregnancy and neonatal outcomes among singletons were also calculated. The neonatal outcomes included gestational age, unadjusted birth weight, very preterm birth (VPTM, <32 weeks’ gestation), preterm birth (PTM, <37 weeks’ gestation), very low birth weight (VLBW, <1500g at birth), low birth weight (LBW, <2500g at birth), high birth weight (HBW, >4500g at birth), and neonatal gender. Z-score was adopted to calculate birthweight adjusted for neonatal gender and gestational age using the following equation: Z-scores=(x-μ)/σ, in which x is the weight of an infant, μ is the mean birthweight at the same gender and same gestational age in the reference group and σ is the standard deviation (SD) of the reference group. Birthweight percentiles and calculation of Z-scores were based on Chinese references singleton newborns stratified by gestational age and neonatal sex (20). Gestational age was calculated by adding 17 days for cleavage-stage embryo transfer and 19 days for blastocyst transfer from embryo transfer date (5).

The basic demographic characteristics of patients, treatment details and outcomes are recorded in the ART database of our center, as required by the Technical Standard for Human Assisted Reproduction issued by the Chinese Ministry of Health (CMOH). Variables extracted for this study included the following: maternal age, maternal body mass index (BMI), type of infertility (primary infertility and second infertility), parity (nulliparous, pluriparous), causes of infertility (female factor, male factor, combined factor, and unexplained infertility), fertilization type (IVF, ICSI), sperm origin (ejaculation and testicular sperm extraction), frozen embryo transfer (FET) cycles rank, embryo quality (good or poor quality) and stage of embryo development (embryo at day 3, 5 or 6).

### Statistical Analysis

The baseline characteristics and neonatal outcomes were described with mean (standard deviation, SD) for continuous variables and percentage for categorical variables. Because more than one cycle from the same patients were included, the potential correlation between repeated embryo transfer cycles by the same patients may influence the outcomes. So we used the generalized estimated equation regression model to explore the association of development stage of embryo, embryo quality, and live birth after controlling potential confounding variables, using the good quality embryo on day 3 group as a reference. Results was reported as unadjusted and adjusted odds ratios (OR) and 95% confidence intervals (CIs). In addition, the generalized estimated equation regressions were also performed to explore the impact of development stage of embryo and embryo quality on gestational age, unadjusted and adjusted birth weight. All statistical analyses were performed by using the two-sided 5% level of significance and the statistical package Stata, Version 12 (StataCorp, College Station, TX).

## Results

Of a total of 6077 single vitrified-thawed embryo transfer cycles, there were 3597 cycles with single good quality embryo vitrified on day 3, 261 cycles with single poor quality embryo vitrified on day 3, 518 cycles with single good quality embryo vitrified on day 5, 120 cycles with single poor quality embryo vitrified on day 5, 926 cycles with single good quality embryo vitrified on day 6, 655 cycles with single poor quality embryo vitrified on day 6. The maternal demographic and treatment characteristics of the included cycles were shown in **Table 1**. The mean maternal age was slightly older for group with embryo transfer at day 3 than group with embryo transfer at day 5/6 and for group with poor quality embryo transfer than group with good quality embryo transfer. A higher proportion of patients underwent their first frozen embryo transfer for group with embryo transfer at day 3 and group with good quality embryo transfer.

**Table 1:** Demographic and treatment characteristics of patients with single frozen-thawed embryo transfer.

	Good quality embryo on day 3	Poor quality embryo on day 3	Good quality embryo on day 5	Poor quality embryo on day 5	Good quality embryo on day 6	Poor quality embryo on day 6
Number of FET cycles	3597	261	518	120	926	655
Maternal age(years), mean ± SD	33.86 ± 5.35	35.04 ± 5.91	31.84 ± 4.19	32.41 ± 4.47	32.31 ± 4.50	33.09 ± 4.74
Maternal BMI(kg/m^2^), mean ± SD	21.88 ± 3.04	21.94 ± 2.88	21.53 ± 2.72	21.30 ± 2.63	21.58 ± 2.92	21.48 ± 2.84
Type of infertility, n(%)						
Primary infertility	1888(52.49)	130(49.81)	277(53.47)	59(49.17)	512(55.29)	332(50.69)
Second infertility	1709(47.51)	131(50.19)	241(46.53)	61(50.83)	414(44.71)	323(49.31)
Parity, n(%)						
Nulliparous	3035(84.38)	206(78.93)	466(89.96)	102(85.00)	832(89.85)	572(87.33)
Pluriparous	562(15.62)	55(21.07)	52(10.04)	18(15.00)	94(10.15)	83(12.67)
Infertility causes, n(%)						
Female	2182(60.66)	143(54.79)	322(62.16)	83(69.17)	562(60.69)	403(61.53)
Male	339(9.42)	24(9.20)	50(9.65)	7(5.83)	86(9.29)	57(8.70)
Mixed	757(21.05)	55(21.07)	110(21.24)	23(19.17)	191(20.63)	136(20.76)
Unexplained	319(8.87)	39(14.94)	36(6.95)	7(5.83)	87(9.40)	59(9.01)
FET cycles rank						
1	2235(62.14)	148(56.70)	260(50.19)	40(33.33)	462(49.89)	282(43.05)
2	917(25.49)	71(27.20)	153(29.54)	48(40.00)	293(31.64)	231(35.27)
≥3	445(12.37)	42(16.09)	105(20.27)	32(26.67)	171(18.47)	142(21.68)
Intracytoplasmic sperm injection fertilization, n(%)						
Yes	2314(64.33)	167(63.98)	337(65.06)	82(68.33)	620(66.95)	423(64.58)
No	1283(35.67)	94(36.02)	181(34.94)	38(31.67)	306(33.05)	232(35.42)
Endometrial preparation program, n(%)						
Natural cycle	788(21.91)	54(20.69)	119(22.97)	26(21.67)	233(25.16)	147(22.44)
Mild Stimulation cycle	1419(39.45)	95(36.40)	231(44.59)	51(42.50)	423(45.68)	278(42.44)
Hormonal replacement cycle	1390(38.64)	112(42.91)	168(32.43)	43(35.83)	270(29.16)	230(35.11)

As shown in **Table 2**, the live birth rate was significantly higher in cycles with single good quality embryo transfer at day 5 (48.65%), single poor quality embryo transfer at day 5 (47.50%), and single good quality embryo transfer at day 6 (36.93%) than cycles with single good quality embryo transfer at day 3 (24.80%). After controlling for maternal age, maternal BMI, type of infertility, parity, infertility causes, FET cycles rank, endometrial preparation program, there are 161% increased odds of a live birth after transfer of single good quality embryo at day 5 (OR=2.61, 95%CI:2.08-3.16, **Table 3**), 152% increased odds of a live birth after transfer of single poor quality embryo at day 5 (OR=2.52, 95%CI:1.77-3.84), 60% increased odds of a live birth after transfer of single good quality embryo at day 6 (OR=1.60, 95%CI:1.37-1.88) compared with transfer of single good quality embryo at day 3. It showed the similar trend in the clinical pregnancy rate. The cycles with single poor quality embryo transfer at day 6 and cycles with single good quality embryo transfer at day 3 were not significantly different in clinical pregnancy rate (36.03% vs. 31.86%) and live birth rate (28.09% vs. 24.80%). The neonatal outcomes of singletons born after single vitrified-thawed embryo transfer at different development stage of embryo and embryo quality were shown in **Table 4**. The adjusted birth weights were significantly higher for transfer single good quality embryo on day 5 (3395.53g) and day 6 (3355.79g) than transfer single good quality embryo on day 3 (3286.99g). Even after controlling for other factors, results from the generalized estimated equation regression showed significant relationship of unadjusted birth weight with development stage of embryo and embryo quality (good quality embryo on day 5 vs. Good quality embryo on day 3: β=108.55, SE=34.89, P=0.002; good quality embryo on day 6 vs. Good quality embryo on day 3: β=68.80, SE=33.75, P=0.041, **Table 5**). The above results were also applied to adjusted birth weight. However, no significant differences were seen in unadjusted birth weight and adjusted birth weight between transfer single poor quality embryo on day 3, 5, 6 and transfer single good quality embryo on day 3.

**Table 2:** Clinical pregnancy outcomes after single frozen-thawed embryo transfer with different embryo quality and development stage.

	Number of cycles	Clinical pregnancy			Live birth		
		rate(%)	OR(95%CI)	Adjusted OR(95%CI)	rate(%)	OR(95%CI)	Adjusted OR(95%CI)
Good quality embryo on day 3	3597	31.86	1	1	24.80	1	1
Poor quality embryo on day 3	261	19.54	**0.52(0.38,0.71)**	**0.53(0.39,0.74)**	14.94	**0.53(0.38,0.76)**	**0.56(0.39,0.80)**
Good quality embryo on day 5	518	56.76	**2.81(2.33,3.38)**	**2.58(2.07,3.03)**	48.65	**2.87(2.38,3.47)**	**2.61(2.08,3.16)**
Poor quality embryo on day 5	120	55.83	**2.70(1.87,3.91)**	**2.50(1.76,3.79)**	47.50	**2.74(1.90,3.96)**	**2.52(1.77,3.84)**
Good quality embryo on day 6	926	44.92	**1.74(1.50,2.02)**	**1.60(1.37,1.87)**	36.93	**1.78(1.52,2.07)**	**1.60(1.37,1.88)**
Poor quality embryo on day 6	655	36.03	**1.20(1.01,1.43)**	1.16(0.97,1.39)	28.09	1.18(0.98,1.43)	1.13(0.94,1.38)

Bold values indicate statistical significance.

**Table 3:** Multiple analysis of factors associated with live birth rate.

	Adjusted OR	95%CI	P value
Good quality embryo on day 3(reference)	1		
Poor quality embryo on day 3	**0.56**	**0.39,0.80**	**0.002**
Good quality embryo on day 5	**2.61**	**2.08,3.16**	**<0.001**
Poor quality embryo on day 5	**2.52**	**1.77,3.84**	**<0.001**
Good quality embryo on day 6	**1.60**	**1.37,1.88**	**<0.001**
Poor quality embryo on day 6	1.13	0.94,1.38	0.200
Maternal age(years)	**0.92**	**0.91,0.93**	**<0.001**
Maternal BMI(kg/m2)	1.01	0.99,1.03	0.323
Type of infertility			
Second infertility vs. primary infertility	0.96	0.85,1.10	0.579
Parity			
Pluriparous vs. nulliparous	**0.80**	**0.65,0.98**	**0.034**
Infertility causes			
Female(reference)	1.07	0.87,1.32	0.520
Male	0.96	0.83,1.12	0.619
Mixed	1.03	0.83,1.27	0.814
Unexplained			
FET cycles rank			
1(reference)	1		
2	0.94	0.82,1.08	0.383
≥3	0.96	0.81,1.14	0.659
Endometrial preparation program,			
Natural cycle(reference)	1		
Mild Stimulation cycle	1.10	0.95,1.29	0.211
Hormonal replacement cycle	1.04	0.89,1.22	0.638

Bold values indicate statistical significance.

**Table 4:** Neonatal outcomes of singletons born after single frozen embryo transfer, stratified by embryo quality and development stage.

	Good quality embryo on day 3	Poor quality embryo on day 3	Good quality embryo on day 5	Poor quality embryo on day 5	Good quality embryo on day 6	Poor quality embryo on day 6	P value
Number of singletons	878	39	247	54	335	177	
Newborn gender, n(%)							0.053
Female	411(46.81)	15(38.46)	106(42.91)	24(44.44)	128(38.21)	90(50.85)	
Male	467(53.19)	24(61.54)	141(57.09)	30(55.56)	207(61.79)	87(49.15)	
Gestational age, mean ± SD	38.35 ± 1.73	38.23 ± 1.63	38.64 ± 1.57	38.56 ± 1.76	38.35 ± 1.78	38.39 ± 1.77	0.111
<32 weeks	9(1.03)	1(2.56)	1(0.40)	1(1.85)	4(1.19)	1(0.56)	0.942
32-37 weeks	57(6.49)	2(5.13)	14(5.67)	2(3.70)	23(6.87)	13(7.34)	
≥37 weeks	812(92.48)	36(92.31)	232(93.93)	51(94.44)	308(91.94)	163(92.09)	
Unadjusted Birthweight, mean ± SD	**3286.99 ± 504.96**	**3266 ± 495.30**	**3395.53 ± 479.42**	**3365.37 ± 622.53**	**3355.79 ± 534.86**	**3242.35 ± 507.01**	**0.014**
Adjusted Birthweight, mean ± SD	**0.28 ± 1.00**	**0.23 ± 0.87**	**0.45 ± 1.06**	**0.42 ± 1.27**	**0.44 ± 1.01**	**0.16 ± 0.98**	**0.022**
<1500g	6(0.68)	0	1(0.40)	1(1.85)	5(1.49)	1(0.56)	0.274
1500-2500g	35(3.99)	1(2.56)	5(2.02)	4(7.41)	11(3.28)	9(5.08)	
2500-4500g	833(94.87)	38(97.44)	239(96.76)	47(87.04)	316(94.33)	166(93.79)	
>4500g	4(0.46)	0	2(0.81)	2(3.70)	3(0.90)	1(0.56)	

Bold values indicate statistical significance.

**Table 5:** Generalized estimated equation model predicting birth weight and gestational age.

	Unadjusted birth weight(g)			Gestational age(weeks)			Adjusted birth weight(g)		
	β	Std.Error	P value	β	Std.Error	P value	β	Std.Error	P value
Good quality embryo on day 3(reference)									
Poor quality embryo on day 3	-5.50	78.58	0.944	-0.15	0.27	0.579	-0.01	0.14	0.974
Good quality embryo on day 5	**114.84**	**34.75**	**0.001**	**0.25**	**0.12**	**0.027**	**0.20**	**0.07**	**0.007**
Poor quality embryo on day 5	82.21	86.38	0.341	0.19	0.25	0.451	0.16	0.18	0.371
Good quality embryo on day 6	**73.76**	**33.47**	**0.028**	-0.03	0.11	0.806	**0.18**	**0.06**	**0.004**
Poor quality embryo on day 6	-27.39	41.87	0.513	0.01	0.15	0.931	-0.06	0.08	0.474
Maternal age(years)	-2.59	3.18	0.417	-0.01	0.01	0.303	-0.01	0.01	0.236
Maternal BMI(kg/m2)	**18.14**	**4.62**	**<0.001**	**-0.04**	**0.02**	**0.014**	**0.06**	**0.01**	**<0.001**
Type of infertility									
Second vs. primary infertility	23.78	27.14	0.381	-0.06	0.09	0.519	0.08	0.05	0.143
Parity									
Pluriparous vs. nulliparous	24.80	47.75	0.603	-0.04	0.15	0.776	0.08	0.09	0.377
Infertility causes									
Female(reference)									
Male	-50.40	41.08	0.220	-0.05	0.16	0.735	-0.11	0.08	0.144
Mixed	-28.03	31.50	0.373	0.05	0.10	0.626	-0.09	0.06	0.149
Unexplained	15.39	44.54	0.730	0.25	0.13	0.056	-0.06	0.10	0.559
FET cycles rank									
1(reference)									
2	8.54	28.23	0.762	0.05	0.10	0.594	-0.01	0.06	0.958
≥3	-6.13	37.61	0.871	0.03	0.12	0.832	-0.02	0.08	0.753
Endometrial preparation program,									
Natural cycle(reference)									
Mild Stimulation cycle	**-104.357**	**29.72**	**<0.001**	**-0.26**	**0.10**	**0.008**	**-0.16**	**0.06**	**0.010**
Hormonal replacement cycle	-3.65	31.21	0.907	-0.13	0.10	0.203	0.06	0.06	0.371

Bold values indicate statistical significance.

## Discussion

Embryo culture period and embryo quality were two important factors influencing live birth outcomes, the purpose of this study was to compare clinical outcomes after blastocyst transfer with cleavage-stage embryo transfer at different grades of embryo quality. To our knowledge, this was the first study to evaluate the pregnancy outcomes and neonatal outcomes in the single vitrified-thaw embryo transfer cycles based on the consideration of both embryo culture period and embryo quality. The present study showed a significant increase in live birth rate and birth weight after transfer of single good quality embryo on day 5 and day 6 compared with transfer of single good quality embryo on day 3 in the vitrified embryo transfer cycles. However, there was no significant difference in birth weight between transfer of single poor quality embryo on day 5 or 6 compared with transfer of single good quality embryo on day 3. This findings indicated the effect of extend embryo culture on birth weight was overcome by transfer of a poor quality embryo in the vitrified-warmed transfer cycles.

Consistent with previously published studies, our study showed that the live birth rate per transfer cycle was significantly higher after blastocyst transfer than cleavage embryo transfer for single good quality embryo (21, 22). Our interesting finding was that the live birth rate for transfer of single poor quality embryo on day 6 and transfer of single good quality embryo on day 3 was equivalent, but transfer of single poor quality embryo on day 5 increased the live birth rate compared with transfer of single good quality embryo on day 3. In the one hand, this study provided clinicians’ important references on how to select embryo to transfer based on consideration of development stage of embryo and embryo quality. Compared with day 3 embryo with good quality, day 5 embryo with poor quality may be the preferred choice, but day 6 embryo with poor quality also was not an inferior choice. In the other hand, this result revealed the superiority of day 5 embryo in embryo implantation over day 6 in single vitrified embryo transfer cycles with poor embryo quality, which was in keeping with some studies (23, 24).

The effect of extend embryo culture on neonatal outcomes in the vitrified transfer cycles has been reported in previous two studies, but the results were conflicting (5, 25). The study performed by Anick et al. presented the lower birth weight after vitrified blastocyst transfer than transfer of vitrified cleavage-stage embryo, but it limited by the exceedingly small sample size (25). Zhang et al. observed that the adjusted birth weight was higher after blastocyst transfer compared with cleavage-stage embryo transfer in the vitrified embryo transfer cycles, but embryo quality was not accounted for in the analysis (5). The present study adds to previous research in that it considers both extended culture and embryo grades, and evaluates the pregnancy and neonatal outcomes after single vitrified embryo transfer with large sample size. Our research found a significantly higher birth weight after blastocyst transfer with good quality embryo compared to cleavage-stage embryo transfer with good quality embryo, but no significant difference in birth weight was seen between blastocyst transfer with poor quality embryo and cleavage-stage embryo transfer with good quality embryo. This findings indicated that the effect of extend embryo culture in birth weight was affected by embryo quality.

While it is known that many embryos on day 3 will not progress to the blastocyst stage, the reasons for this are still to be elucidated. The exact mechanism of the association between live birth rate/birth weight and culture duration remains unknown. Further evidence provided new insight that the early embryonic development stage is vulnerable and sensitive to its environmental conditions such as *in vitro* culture, any perturbation in this process would force embryos make adaptation *via* epigenetic alternations, leading to alternations in fetal growth trajectory and hence live birth rate and birth weight (26, 27).

Previous studies have explored the individual effect of each variable on IVF outcomes. Some investigators have shown that expansion stage is an effective predictor of implantation (28, 29). Others have observed a strong association between grade of ICM and IVF success rate (30, 31). Noteworthy is that TE stage has been reported to be positively associated with implantation, and its predictive strength exceeded that of ICM for selecting the best blastocyst (6, 32). However, others declare that no relationship between TE stage and pregnancy outcomes was observed (30, 33). This controversy will persist until conclusive evidence is provided by adequately powered randomized controlled trials.

The main strength of present study was eliminating the possibility effect of ‘vanishing twins’ in clinical outcomes by including only single embryo transfer. The second strength was that the same embryo culture medium was used during the study period, which avoiding the adverse influence of different culture media on pregnancy outcomes and neonatal outcomes (34, 35). In addition, the embryo quality was assessed by the same team of embryologists in the same center using the uniform standards, which could reduce some bias from observers. However, there were also several limitations in our study. First, although a single center study contributed to the unchanged culture media and embryo evaluation, the conclusion from our study need to be verified in the further prospective multi-center study. Second, we could not adjust for some potential confounding variables in the analysis, such as smoking and environment exposure, because these variables were not available in this study.

## Conclusion

In conclusion, we compared the live birth rate and neonatal outcomes after blastocyst transfer with cleavage-stage embryo transfer at different grades of embryo quality. We found that a significant increase in live birth rate and birth weight after transfer of single good quality embryo on day 5 and day 6 compared with transfer of single good quality embryo on day 3 in the vitrified embryo transfer cycles. But there was no significant difference in birth weight between transfer of single poor quality embryo on day 5 or 6 compared with transfer of single good quality embryo on day 3. Our results indicated that embryo quality needs to be given full consideration when assessing the clinical outcomes after blastocyst transfer versus cleavage embryo transfer. However, further prospective studies are needed to verify these findings.

## Data Availability Statement

The raw data supporting the conclusions of this article will be made available by the corresponding authors, without undue reservation.

## Ethics Statement

The studies involving human participants were reviewed and approved by the Ethics Committee of the Shanghai Ninth People’s Hospital. Written informed consent for participation was not required for this study in accordance with the national legislation and the institutional requirements.

## Author Contributions

NW, QZ and YW conceived and designed the study. XZ and MM collected patient data. All authors contributed to the article and approved the submitted version.

## Funding

This work was supported by the National Natural Science Foundation of China (no. 81801527 and no. 81903324).

## Conflict of Interest

The authors declare that the research was conducted in the absence of any commercial or financial relationships that could be construed as a potential conflict of interest.
